# Exploring anti-cancer activities of epidermal growth factor-immobilized polymeric nanoparticles

**DOI:** 10.1080/14686996.2025.2463316

**Published:** 2025-02-06

**Authors:** Shota Yamamoto, Chia-Jung Chang, Masao Kamimura, Jun Nakanishi

**Affiliations:** a Research Center for Macromolecules & Biomaterials, National Institute for Materials Science (NIMS), Tsukuba, Ibaraki, Japan; b Graduate School of Advanced Engineering, Tokyo University of Science, Katsushika-ku, Tokyo, Japan; c Graduate School of Advanced Science and Engineering, Waseda University, Shinjuku-ku, Tokyo, Japan; d Research Center for Autonomous Systems Materialogy (ASMat), Institute of Integrated Research (IIR), Institute of Science Tokyo (Science Tokyo), Yokohama, Kanagawa, Japan

**Keywords:** Anti-cancer drugs, EGF-nanoparticle conjugates, polymeric micelles, triple-negative breast cancer

## Abstract

Cytotoxic agents targeting the epidermal growth factor receptor (EGFR) exhibit significant potential for cancer therapy as EGFR is overexpressed in various cancers. As alternatives to conventional EGFR inhibitors (EGFRi), which exert side effects on non-cancer cells, EGF-immobilized gold nanoparticles exhibit selective cytotoxicity in EGFR-overexpressing cancer cells by locally enhancing EGFR activation and modulating signal transduction through a signal condensation mechanism. However, considering real therapeutic applications, it is important to confirm that the same principle can be applied to polymeric nanoparticles, which are more suitable carriers owing to their biodegradability and biocompatibility, remains unclear. Therefore, in this study, we aimed to investigate the anti-cancer activities of EGF-conjugates with two kinds of polymeric nanoparticles: polystyrene nanoparticles and polymeric micelles. Initial mechanistic studies on phosphorylation signaling and cholesterol depletion revealed that EGF-polystyrene nanoparticles exhibited cytotoxicity against human cervical adenocarcinoma HeLa cells via local enhancement of EGFR activity in membrane rafts. Moreover, EGF-polymeric micelles exerted selective anti-cancer effects against EGFRi-resistant MDA-MB468 refractory triple-negative breast cancer cells after optimization of particle size. These results suggest that the unique anti-cancer effects of EGF nanoparticles are not dependent on the carrier platform. Furthermore, EGF nanoparticles exhibited high cytotoxicity against cancer cells responding poorly to conventional EGFR-targeted anti-cancer drugs, showing potential for future medical applications.

## IMPACT STATEMENT

Epidermal growth factor (EGF)-immobilized polymeric nanoparticles demonstrated anti-cancer activity in cancer cells that respond poorly to conventional EGF receptor-targeted anti-cancer drugs, indicating promising potential for medical applications.

## GRAPHICAL ABSTRACT

**Figure:**
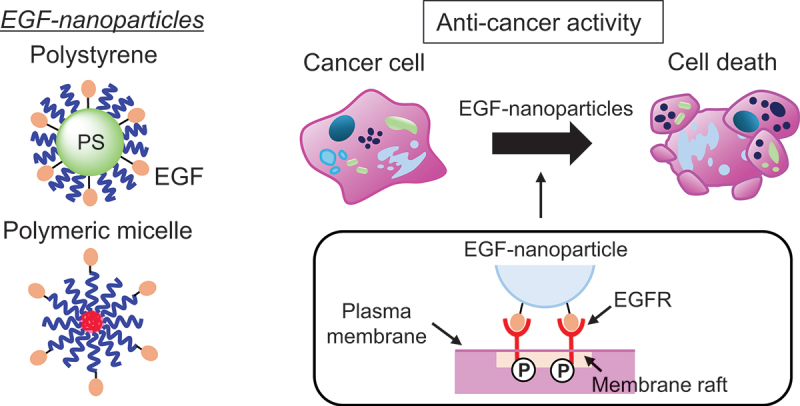

## 1. Introduction

Epidermal growth factor (EGF) receptor (EGFR) is a transmembrane protein overexpressed in various cancers such as lung cancer [1], breast cancer [2], head and neck squamous cell carcinoma [3]. EGFR plays critical roles in the regulation of various cellular processes, such as cell proliferation, differentiation, and survival [4]. Its overexpression and aberrant activation are closely associated with oncogenesis, making it a promising target for cancer therapies [5,6]. Cancer therapies can be broadly classified into two categories: tyrosine kinase inhibitor and monoclonal antibody therapies [7]. Tyrosine kinase inhibitors, such as gefitinib, erlotinib, and afatinib, are small molecules that inhibit the kinase activity of EGFR by competing with adenosine triphosphate (ATP to bind to the intracellular kinase domain [8]. These inhibitors show considerable efficacy for the treatment of patients with non-small cell lung cancer with activating EGFR mutations [9]. Cetuximab and panitumumab are monoclonal antibodies that target the extracellular domain of EGFR, preventing ligand binding and subsequent receptor activation [10]. These antibodies are particularly effective in treating colorectal and head and neck cancers [11]. Two typical anti-cancer drugs targeting EGFR show efficacy against cancer cells by inhibiting EGFR signaling. Despite the recent advancements, development of EGFR-targeted therapies remains a challenge. Due to the important role of EGFR in maintaining homeostasis in the skin, nails, and hair, side effects are often observed with the use of EGFR inhibitors [12]. Additionally, primary and acquired resistance to EGFR inhibitors complicates treatment [13]. Resistance mechanisms include secondary mutations in EGFR, activation of alternative signaling pathways, and phenotypic changes, such as epithelial–mesenchymal transition [14]. Cancer cells with genetic mutations or receptor heterogeneity, such as those in triple-negative breast cancer, exhibit resistance to EGFR inhibitors [15]. Therefore, new strategies and EGFR-targeting anti-cancer drugs are urgently needed for effective cancer treatment with low side effects.

EGF-immobilized gold nanoparticles (NPs) serve as a new candidate for anti-cancer drugs targeting EGFR. EGF is an endogenous protein that induces signal transduction by interacting with EGFR, thereby promoting cell growth and differentiation [16]. However, when EGF is immobilized on gold NPs, its effect on cancer cells changes from promoting proliferation to inducing apoptosis [17–20]. We previously demonstrated that this unique apoptosis-inducing activity is due to signal condensation in membrane rafts, where the effect of EGF is confined by NPs [21]. As anti-cancer activity via signal condensation highly depends on the EGFR expression levels in target cells, these NPs exhibit negligible cytotoxicity in normal cells [19]. Furthermore, we demonstrated that introducing a mutated EGF onto gold NPs enhances cytotoxicity by preferentially activating a clathrin-independent endocytosis pathway [22]. This property is more beneficial for anti-cancer therapeutics than conventional EGFR inhibitors for minimal side effects. However, to date, anti-cancer activities of EGF-NPs have only been investigated using gold NPs as EGF carriers, as it is easy to control their diameter, shape, and surface functionalities for the fundamental analysis of their impacts on cellular cytotoxicity and intracellular signaling [17]. However, for practical therapeutic applications, polymeric NPs, such as those based on polymeric micelles and biodegradable polymers, are more favorable owing to their capability to design pharmacokinetics and biocompatibility from the chemical structure. These NPs can be efficiently delivered to tumor tissues using the enhanced permeability and retention effect [23]. Several studies have reported the application of polymeric NPs functionalized with EGF- or EGFR-binding peptides to deliver anti-cancer drugs to cancer cells using pilot ligands as targeting molecules [24–26]. However, to directly use EGF-NPs as novel anti-cancer agents, their unique cytotoxicity via signal condensation needs to be investigated using polymeric platforms.

In this study, we developed two kinds of polymeric platforms, namely polystyrene (PS) and polymeric micelles, to explore the anti-cancer effects in their nanoparticles. To evaluate the cytotoxicity induced by EGF polymeric nanoparticles, we first investigated the cellular responses, mechanism of action, and anti-cancer activities of EGF-NPs with PS as a carrier (EGF-PSNPs) using the HeLa human cervical adenocarcinoma cells. Additionally, to explore their potential for medical applications, EGF-polymeric micelles, tethering EGF on polymeric micelles, were administered to MDA-MB468 human triple-negative breast cancer cells, which are typically resistant to EGFR inhibitors, to investigate their time-dependent anti-cancer activities and impact of particle size on these activities. The established NPs were effective against refractory cancer cells, showing potential as novel anti-cancer agents.

## 2. Materials and methods

### 2.1. Reagents and chemicals

EGF (Sigma, USA), amino-terminated poly(ethylene glycol) (PEG; Mw: 5,000; NOF corporation, Japan), polylactide (PLA)-PEG-COOH (PLA5k-PEG5k; Creative PEGWorks, USA), PS Latex NPs (PSNPs; 0.02 μm; ThermoFisher, USA), green fluorescent PSNPs (0.02 μm; ThermoFisher) and red fluorescent PSNPs (0.02 μm; ThermoFisher) were commercially purchased and used without further purification.

### 2.2. Preparation of EGF-PSNPs

PSNPs (0.0547 μM), green fluorescent PSNPs (0.0547 μM), or red fluorescent PSNPs (0.0547 μM) were functionalized in a phosphate-buffered saline (PBS) solution containing amino-terminated PEG (1.10 μM), EGF (0.274 μM), and 1-ethyl-3-(3-dimethylaminopropyl)carbodiimide hydrochloride (EDC; 1.37 μM) using a shaking mixer at 4°C overnight. Then, functionalized PSNPs were washed with PBS and centrifuged at 271,000 × *g* for 30 min at 4°C (three times) to obtain EGF-PSNPs. EGF concentration in the supernatant was measured via direct enzyme-linked immunosorbent assay (ELISA) using the rabbit anti-EGF (Abcam, UK) and anti-rabbit IgG horseradish peroxidase conjugate (Sigma) antibodies, with 3,3‘,5,5’-tetramethylbenzidine (KPL, USA) as the substrate. Finally, the signals were detected using a plate reader (Bio-Rad, USA).

### 2.3. Preparation of egf-polymeric micelles

The preparation method for 50 nm EGF-polymeric micelles is described as a representative example. PLA5k-PEG5k (3 mg; 3.0 × 10^−7^ mol) was dissolved in 3 mL acetonitrile. The polymer solution was mixed with 10 mL 2-(*N*-morpholino)ethanesulfonic acid buffer (MES; Sigma) and stirred at room temperature for 18 h. Then, the polymer micelle solution was evaporated under controlled pressure for 30 min to remove acetonitrile and concentrated thrice using a centrifugal concentrator (Vivaspin 20; MWCO: 30000; Sartorius, Germany) at 800 × *g* for 10 min. Polymeric micelles were functionalized in PBS containing EDC (120 mm), EGF (3.3 μM), and sulfo-NHS (120 mm). The mixture was gently shaken at 4°C overnight. EGF-polymeric micelles were washed with PBS and centrifuged at 800 × *g* for 10 min at 4°C (five times) using a centrifugal concentrator (Amicon Ultra; MWCO: 100,000; Merck, Germany). Polymeric micelles with 35 nm, 100 nm, and 150 nm diameter were prepared using 2 mL acetonitrile and 10 mL MES buffer, 4 mL acetonitrile and 8 mL MES buffer, and 4 mL acetonitrile and 7 mL MES buffer, respectively.

### 2.4. Characterization of EGF-PSNPs and egf-polymeric micelles

Diameters and particle size distributions of EGF-PSNPs and EGF-polymeric micelles were measured via dynamic light scattering (Otsuka Electronic Co., Ltd., Japan). The prepared EGF-PSNPs and EGF-polymeric micelle solutions were placed in disposable cuvettes. All measurements were taken at a scattering angle of 90° at room temperature.

### 2.5. Cell culture

Cell lines were purchased from the American Type Culture Collection (ATCC) and RIKEN BRC. HeLa cells (cervical cancer cells; RIKEN BRC; RCB0007) were cultured in the minimum essential medium eagle (MEM) supplemented with 10% heat-inactivated fetal bovine serum (FBS; Biowest, France), 100 U/mL penicillin, and 100 mg/mL streptomycin at 37°C in a humidified atmosphere containing 5% CO_2_. MDA-MB468 cells (triple-negative breast cancer cells; ATCC; HTB-132) were cultured in the Dulbecco’s modified Eagle’s medium (DMEM) supplemented with 10% heat-inactivated fetal FBS, 0.2% MycoZap (Lonza, Switzerland), 100 U/mL penicillin, and 100 μg/mL streptomycin at 37°C in a humidified atmosphere containing 5% CO_2_. EA.hy926 cells (umbilical vein cells; ATCC; CRL2922) were cultured in DMEM supplemented with 10% heat-inactivated FBS, 100 U/mL penicillin, and 100 mg/mL streptomycin at 37°C in a humidified atmosphere containing 5% CO_2_. MCF10A cells (breast epithelial cell; ATCC; CRL10317) were cultured in the mammary epithelial cell growth medium bullet kit (Lonza) at 37°C in a humidified atmosphere containing 5% CO_2_.

### 2.6. Cell ELISA

HeLa cells were seeded in a 96-well plate at a density of 2.0 × 10^4^ cells/well for 24 h and serum-starved in 50 µL of medium for 4 h. Then, the cells were exposed to EGF-PSNPs (10 nM) and soluble EGF (1000 ng/mL) for 5 min at 37°C and fixed with 4% paraformaldehyde (Wako, Japan) in PBS for 20 min at room temperature. After washing with PBS, the cells were permeabilized with 0.5% Triton-X (Sigma) in PBS for 20 min, blocked with 2% bovine serum albumin in PBS, and incubated with phospho-extracellular signal-regulated kinase (ERK)-1/2 (T202/Y204) rabbit monoclonal antibodies (1:1000; Cell Signaling Technology, USA), followed by incubation with anti-rabbit IgG alkaline phosphatase (1:3000, Sigma) in PBS containing 1% bovine serum albumin for 1 h at room temperature. The bound antibodies were detected using 4-nitrophenyl phosphate disodium salt hexahydrate (Sigma) as a substrate.

### 2.7. Apoptosis assay

HeLa cells were seeded in a 6-well plate at a density of 2.0 × 10^4^ cells/well, incubated at 37°C for 24 h, and serum-starved at 37°C for 4 h. After treatment with EGF-PSNPs (9 nM), soluble EGF (1000 ng/mL), PEG-PSNPs (9 nM) or MEM for 4 h at 37°C, the cells were washed and incubated in with MEM containing 10% FBS for 68 h at 37°C. After detaching the cells using trypsin-EDTA, the suspension was centrifuged to remove the supernatant. The pellet was resuspended using the Annexin V-Fluorescein Isothiocyanate (FITC) Apoptosis Detection Kit (Nacalai, Japan) containing the annexin V-FITC conjugate and propidium iodide (PI). After incubation at room temperature for 15 min, the stained cells were detected using the cell analyzer SP6800 (Sony, Japan).

### 2.8. Colocalization study of membrane rafts and EGF-PSNPs

HeLa cells were seeded onto 3.5 cm glass bottom dish at 2.0 × 10^4^ cells/dish for 24 h at 37°C. Cells were incubated with cholera toxin subunit B (recombinant) with Alexa Fluor 488 (1:100; ThermoFischer) to stain GM1 for 1 h at 4°C. After washing with ice-cold PBS, HeLa cells were treated with red fluorescent EGF-PSNPs for 5 min at 4°C. The cells were washed with ice-cold PBS and fixed with 4% paraformaldehyde in PBS for 10 min at 4°C. The GM1 and EGF-PSNPs were visualized by an Olympus BX-51 microscope equipped with a LUMPLFLN 40×W lens (Olympus, Japan) and MD695 cooled CCD camera (Hamamatsu photonics, Japan) controlled by MetaMorph (Molecular devices, USA).

### 2.9. Cellular uptake of EGF-PSNPs

HeLa cells were seeded onto 3.5 cm glass bottom dish at 2.0 × 10^4^ cells/dish for 24 h at 37°C. The green fluorescent EGF-PSNPs were added to the cells and incubated for 1 h at 37°C. After washing the cells with Hank’s Balanced Salt Solution (HBSS), EGF-PSNPs were visualized by confocal laser microscopy by using an IX81-PAFM fluorescence microscope (Olympus) equipped with a disk scan unit (CSU-10, Yokokawa, Japan) an Andor laser combiner (Oxford Instruments, UK), an CMOS camera SONA (Oxford Instruments) controlled by MetaMorph.

### 2.10. Cell viability assay

MDA-MB468, MCF10A, and EA.hy926 cells were cultured in 96-well plates. After overnight incubation at 37°C, the cells were treated with EGF-polymeric micelles, polymeric micelles without EGF, or medium for 3, 6, 12, 24, 48, and 72 h. Then, the medium was gently removed, and the cells were washed twice with PBS. Cell counting kit-8 solution (Dojindo; 100 µL) was added to each well, and cell viability was assessed via cell proliferation assay, according to the manufacturer’s protocol. After 2-h incubation, absorbance was measured at 450 nm using a microplate reader (Multiskan FC; Thermo Fisher).

### 2.11. Statistical analyses

Cell ELISA, apoptosis assay, and cell viability assay results were evaluated based on the average and standard deviation of at least three different experiments. Statistical differences were analyzed using the Student’s *t*-test.

## 3. Results and discussion

### 3.1. Preparation and characterization of EGF-PSNPs

**Figure 1.**
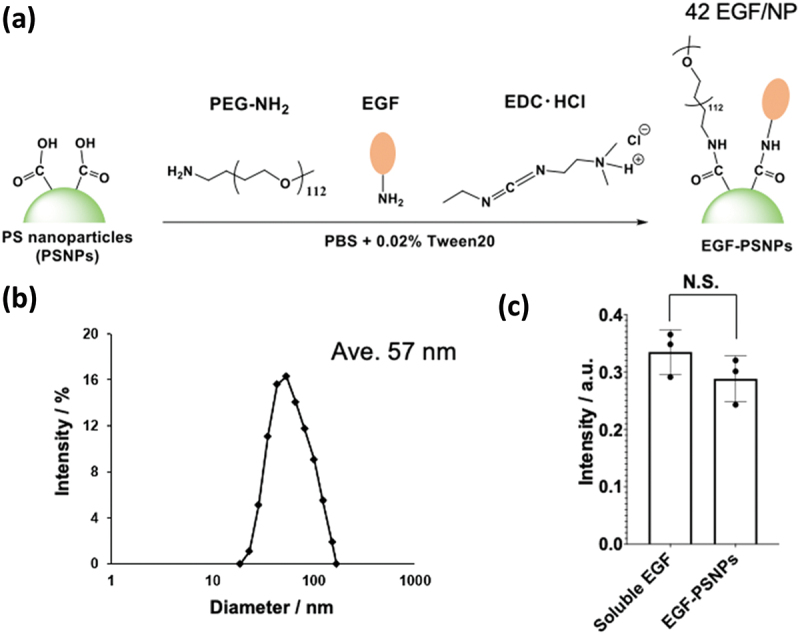
Synthesis and characterization of the epidermal growth factor (EGF)-polystyrene nanoparticles (PSNPs). (a) EGF-PSNPs synthesis scheme. (b) Dynamic light scattering analysis of EGF-PSNPs. (c) Activation of extracellular signal-regulated kinase (ERK) by soluble EGF (1 μg/mL) and EGF-PSNPs (9 nM). Data are represented as the mean ± standard deviation (SD) of three independent experiments. Statistical differences were analyzed via Student’s *t*-test.

To investigate the generality of the anti-cancer effects of EGF-NPs, we prepared EGF-PSNPs using PS Latex beads by functionalizing the carboxyl groups on their surfaces with amide coupling (Figure 1a). For this study, we selected PSNPs with a carrier size of 20 nm, at which gold NPs has been shown to induce apoptosis in cancer cells [21]. The two EGF molecules and PEG brush were immobilized on the PS particle surface. PEG chains are essential not only to ensure stable particle dispersion in aqueous solutions but also to prevent non-specific protein adsorption on the particle surface. To prevent the aggregation of EGF-PS NPs, molar ratio of EGF to PEG was set at 1:4 for surface modification. The developed EGF-PSNPs were washed with repeated centrifugation to remove the unreacted EGF, PEG, and EDC. PEG-PSNPs without EGF, which were used for control experiments, were prepared by immobilizing only PEG. Diameters of EGF-PSNPs and PEG-PSNPs were measured via dynamic light scattering. Successful functionalization of PSNPs was confirmed by the increased diameters of EGF-PSNPs and PEG-PSNPs compared to the initial 20 nm diameter of PSNPs. The prepared EGF-PSNPs and PEG-PSNPs exhibited a particle size of approximately 57 nm (Figures 1b and S1). A previous study showed that EGF-NPs with 50–200 nm size induced apoptosis [17]. Based on the change in the EGF concentration of the solution before and after the reaction with PSNPs, average number of EGF molecules conjugated to a single NP was found to be 42. We previously reported that the density of EGF modification, 13–70 molecules/particle, does not significantly affect its apoptosis-inducing activity [19]. Therefore, we expected our prepared EGF-PS-NPs had potential to exhibit anti-cancer activity through apoptosis based on the particle diameter and surface EGF density. To evaluate the biological activity of the prepared EGF-PS NPs, phosphorylation of ERK, a major downstream protein involved in EGFR signaling, was monitored via ELISA. HeLa cells were treated with EGF-PSNPs and 1 μg/mL soluble EGF for 5 min. Phosphorylation levels of EGF-PS NPs were comparable to those of soluble EGF (Figure 1c). Notably, EGF-gold NPs with similar size and amount of EGF immobilization as EGF-PSNPs exhibited ERK activation comparable to that of soluble EGF [21]. These results suggest that changing the carrier from gold NPs to polymer NPs has no significant effect on EGF activities.

### 3.2. Anti-cancer activities of EGF-PSNPs

**Figure 2.**
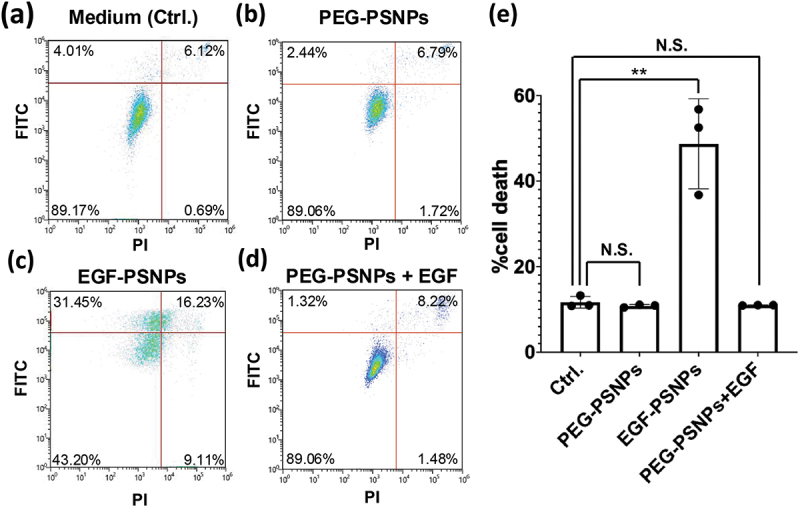
Apoptotic activities of the medium, EGF-PSNPs, poly(ethylene glycol) (PEG)-PSNPs, and PEG-PSNPs + soluble EGF against HeLa cells. Apoptotic cells were detected via annexin V and propidium iodide (PI) staining for 72 h. Flow cytometry result of (a) medium (Ctrl.), (b) PEG-PSNPs, (c) EGF-PSNPs and (d) the co-administration of soluble EGF and PEG-PSNPs. (d) Cytotoxicities of the medium, EGF-PSNPs, PEG-PSNPs, and PEG-PSNPs + soluble EGF. Data are represented as the mean ± SD of at least three independent experiments. Statistical differences were analyzed via Student’s *t*-test (**p < 0.01).

To investigate the anti-cancer activities of EGF-PSNPs, HeLa cells were incubated with EGF-PSNPs, soluble EGF, PEG-PSNPs with soluble EGF, and medium for 4 h. Annexin V-FITC and PI were used to stain the dead cells that underwent apoptosis and necrosis after 72 h of incubation. Stained HeLa cells were analyzed using a flow cytometer (Figure 2a–d). Then, the rate of cell death was calculated as the sum of early apoptotic cells stained with FITC, late apoptotic cells stained with both FITC and PI, and necrotic cells stained with PI (Figure 2E). As expected, the administration of EGF-PSNPs induced cell death in approximately 50% of HeLa cells after 72 h of incubation (Figure 2c,e). Additionally, no apoptotic activity was observed with PEG-GNPs without the EGF ligand as the population of apoptotic cells was nearly identical to that observed after treatment with the medium (Figure 2a,b,e). Notably, despite the co-administration of soluble EGF and PEG-PSNPs, HeLa cells did not undergo apoptosis (Figure 2d,e). As normal EGF induces cell proliferation, this result suggests that the immobilization of EGF on PSNPs switches its effect to induce cell death.

**Figure 3.**
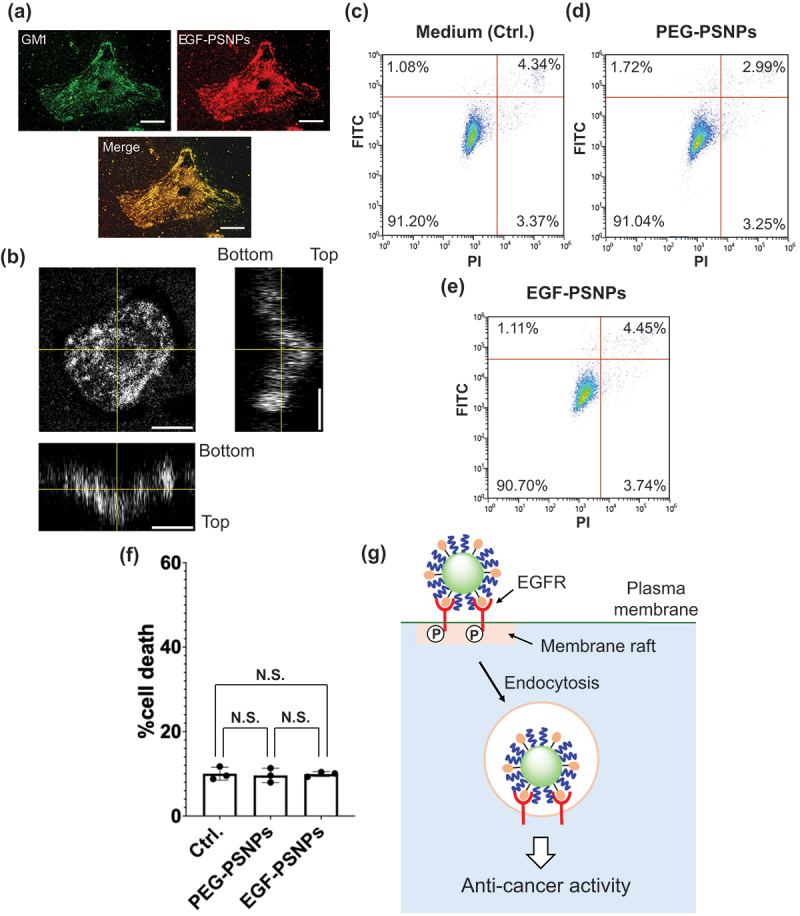
(a) Fluorescence analysis of GM1 (green), EGF-PSNPs (red) and merge image 5 min after reaction. Scale bars: 20 μm. (b) Confocal image of EGF-PSNPs 1 hour after reaction. Scale bars: 20 μm. (c-f) Flow cytometry results after membrane raft destruction by β-cyclodextrin on the apoptotic activities of (c) medium, (d) PGF-PSNPs, and (e) EGF-PSNPs. Apoptotic cells were detected via annexin V and PI staining for 72 h. (f) Cytotoxicity of medium, EGF-PSNPs, and PEG-PSNPs to HeLa cells after treatment with β-cyclodextrin. Data are represented as the mean ± SD of at least three independent experiments. Statistical differences were analyzed via Student’s *t*-test. (g) Expected mechanism by which EGF-PSNPs acquire anti-cancer activity.

To investigate the mechanisms underlying the anti-cancer activities of EGF-PSNPs, we focused on membrane rafts on the cell membrane. Membrane rafts are cholesterol- and sphingolipid-enriched membrane nanodomains (80–100 nm) on the plasma membrane that serve as reaction centers for EGFR signaling [27,28]. We hypothesized that the anti-cancer effect of EGF-NPs with PS as a carrier occurs by regulating the EGFR activity in membrane rafts. We initially investigated the interaction between EGF-PSNPs and HeLa cells using fluorescence microscopy. EGF-PSNPs were prepared by using commercial red fluorescent PSNPs for their visualization. Simultaneously, membrane rafts were stained with Alexa Fluor 488-labeled cholera toxin subunit B, which binds to GM1, a glycosphingolipid specific to rafts. Figure 3a shows the fluorescence images of PSNPs and GM1 after a 5-minute incubation. The merged image of PSNPs and GM1 signals clearly indicates that EGF-PSNPs interact with membrane rafts during the initial reaction on plasma membrane. To further investigate the intracellular localization of EGF-PSNPs, their distribution was tracked following a 1-hour incubation. Figure 3b presents both a top-view fluorescence image and cross-sectional fluorescence images of the green fluorescent PSNPs. Most of the EGF-PSNPs were observed in the cytoplasm, indicating that they were internalized through EGF receptor-mediated endocytosis. Next, to investigate the relationship between membrane rafts and the cytotoxicity induced by EGF-PSNPs, we disrupted the membrane rafts by depleting cholesterol from the cell membrane in HeLa cells using 5 mm *β*-cyclodextrin. Then, the cells were treated with EGF-PSNPs, PEG-PSNPs, and medium and incubated for 72. Subsequently, HeLa cells were stained with annexin V-FITC and PI. Figure 3c–e show the flow cytometry results for HeLa cells treated with each sample. Then, the rate of cell death was calculated by summing early apoptotic cells stained with FITC and PI (Figure 3f). Notably, EGF-PSNPs exhibited approximately 10% cytotoxicity in HeLa cells treated with β-cyclodextrin, resulting in the loss of their unique anticancer activity (Figure 3e,f). Moreover, most HeLa cells treated with the medium and PEG-PSNPs were not stained by annexin V-FITC and PI, indicating that β-cyclodextrin did not affect the cell viability (Figure 3c,d,f). These results suggest that the anti-cancer activities of EGF-PSNPs are not due to NP accumulation within cells but instead mediated through specific EGFR signaling via membrane rafts. Figure 3g shows the reaction mechanism of EGF-PSNPs. When administered to HeLa cells, EGF-PSNPs interact with EGFR within membrane rafts during the initial reaction phase. Subsequently, the particles are internalized via receptor-mediated endocytosis, thereby acquiring anti-cancer activity. We previously reported the role of membrane rafts in the apoptosis-inducing activity of EGF conjugated with gold NPs [21]. Furthermore, EGF gold NPs enhance cytotoxicity by preferentially activating the clathrin-independent endocytosis pathway [22]. It has also been reported that the apoptotic activity of EGF gold NPs is acquired by sustaining EGFR activation within early endosomes for an extended period following endocytosis [18]. Eventually, the anti-cancer activity of EGF nanoparticles depends on the interaction with EGFR mediated by membrane rafts and the subsequent membrane trafficking, both of which are critical key steps. Therefore, our findings suggest that EGF induces cancer cell death via similar mechanisms of gold NPs even when immobilized on different polymer carriers.

### 3.3. Preparation and characterization of egf-polymeric micelles

**Figure 4.**
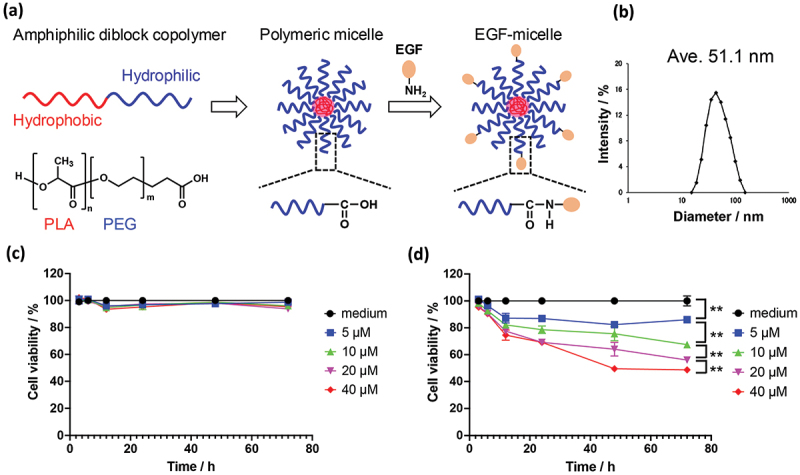
Preparation and characterization of EGF-polymeric micelles. (a) EGF-polymeric micelle preparation scheme. (b) Dynamic light scattering analysis study of EGF-polymeric micelles. (c) Dose-dependent curves of the growth inhibitory activities of (c) polymeric micelles without EGF and (d) EGF-polymeric micelles against MDA-MB468 cells. Here, we discuss the dose-dependency of the concentration of diblock copolymer. MDA-MB468 cells were treated with polymeric micelles without EGF or EGF-polymeric micelles for 72 h. Cell viability was assessed based on the growth inhibitory effects on cells relative to those on cells treated with the medium. Data are represented as the mean ± SD of at least three independent experiments. Statistical differences were analyzed via Student’s *t*-test (**p < 0.01).

EGF-NPs are beneficial for novel cancer treatment strategies aimed at enhancing rather than inhibiting EGFR activity. In this study, we applied the concept of cancer treatment using EGF-NPs to polymeric micelles. Polymeric micelles are composed of block copolymers with polylactic acid (hydrophobic polymer) and PEG with terminal carboxyl groups (hydrophilic polymer), which are highly biocompatible (Figure 4a) [29]. Subsequently, EGF molecules were immobilized on the surface of the prepared polymeric micelles via condensation reaction to yield the EGF-polymeric micelles. Unreacted EGF and condensing agents were removed via repeated ultrafiltration. EGF-polymeric micelles exhibited an average particle size of approximately 51 nm and were monodisperse (Figure 4b). Furthermore, the prepared polymeric micelles maintained a stable structure, with no change in particle size observed, even after three days (Figure S2). Larger (150 nm) EGF-polymeric micelles were obtained by increasing the ratio of the organic solvent during the self-assembly of block copolymers (Figure S3). This is because increased amount of organic solvent strengthens the interactions between the hydrophobic blocks, leading to the formation of micelles with larger cores. The resulting 50 nm polymeric micelles without EGF and EGF-polymeric micelles were administered to MDA-MB468 triple-negative breast cancer cells, and the concentration and time profiles of their anti-cancer activities were evaluated using a cell viability/cytotoxicity assay. Here, we discuss the dose-dependency of the concentration of diblock copolymer. Different micelle doses (5, 10, 20, and 40 μM at diblock copolymer concentration ) were used, and cell viability was measured at 3, 6, 12, 24, 48, and 72 h post-administration. Figure 4c shows the assay results for the polymeric micelles without immobilized EGF. Even at a high concentration of 40 μM, the polymeric micelles did not exhibit cytotoxicity, and the cell viability was comparable to that in the group treated with the medium for 72 h. In contrast, EGF-polymeric micelles decreased the viability of MDA-MB468 cells over time at all concentrations (Figure 4d). Increasing concentrations of polymeric micelles further increased their anti-cancer activities. Furthermore, MDA-MB468 cells exhibited a similar level of cytotoxicity in response to EGF-PSNPs as to EGF-polymeric micelles (approximately 50% cytotoxicity at 72 h). This result strongly suggests that the anti-cancer activities of EGF-NPs can be induced using various biocompatible materials, including polymeric micelles, regardless of the particle type. Therefore, EGF-polymeric micelles can be used to treat triple-negative breast cancer cells, which is typically difficult to treat with conventional EGFR-targeting drugs. Cell viability tests revealed that the larger 150 nm EGF-polymeric micelles exhibited high cytotoxicity (Figure S4). However, even polymeric micelles without EGF reduced the viability of MDA-MB468 cells (Figure S5). Polymeric micelles with size >150 nm exhibit cytotoxicity due to lysosomal dysfunction [30] and physical stress from escaping endosomes [31]. Although large EGF polymeric micelles show enhanced anti-cancer efficacy, they also cause pronounced side effects. To further investigate the effect of particle size on the anti-cancer activity of EGF-polymeric micelles, we prepared smaller (35 nm) and larger (100 nm) EGF-polymeric micelles in addition to the 50 nm polymeric micelles. These particles were administered to MDA-MB468 cells, and cell viability was evaluated after 24 and 72 hours (Figure S6). The 35 nm EGF-polymeric micelles exhibited minimal anti-cancer activity against MDA-MB468 cells, whereas the 100 nm EGF-polymeric micelles showed cytotoxicity comparable to that of the 50 nm polymeric micelles. Next, we investigated the effect of 50 nm EGF-polymeric micelles on normal cells. In addition to the MDA-MB468 triple-negative breast cancer cells, we used the MCF10A normal mammary epithelial cells and normal EA.Hy926 vascular endothelial cells for analysis. Cytotoxicity levels in MCF10A and EA.Hy926 cells were very low and nearly equivalent to the background levels observed in the medium-treated cells (Figures S7 and S8). Negligible cytotoxicity was observed in normal cells (MCF10A and EA.Hy926), which is advantageous for anti-cancer drugs. Moreover, no cytotoxicity was observed in the MCF10A normal mammary cells, indicating the potential of the EGF-polymeric micelles to decrease the side effects on triple-negative breast cancer cells during treatment. Our findings suggest that 50–100 nm EGF-polymeric micelles are cytotoxic to cancer cells. Furthermore, anti-cancer activities of EGF-NPs can be extended not only to polymeric micelles but also to other bioadaptive materials, such as phospholipids and nanogels.

## 4. Conclusion

In this study, we explored the anti-cancer effects of EGF-immobilized NPs using PSNPs and polymeric micelles as carrier platforms. EGF-PSNPs exhibited anti-cancer activity against HeLa human cervical adenocarcinoma cells, but PEG-PSNPs without EGF were non-cytotoxic. Subsequent analysis revealed that cytotoxicity was induced by membrane raft-mediated EGF signaling. These results suggest that the anti-cancer effects of EGF-NPs are not dependent on the carrier. Moreover, EGF-polymeric micelles exerted cytotoxic effects on MDA-MB468 human triple-negative breast cancer cells, which are generally resistant to EGFR-targeting anti-cancer drugs. Notably, high concentrations of polymeric micelles exerted no cytotoxic effects on normal cells but enhanced the anti-cancer effects on cancer cells. However, increase in the particle size of polymeric micelles, including carrier polymeric micelles without EGF, induced cytotoxicity. This result underscores the importance of maintaining the particle size within a specific range for low-side-effect cancer therapy. Overall, this study highlights EGF-NP conjugates as novel anti-cancer drugs.

## Supplementary Material

Supplemental Material
