# Supplementary material for: Exploring anti-cancer activities of epidermal growth factor-immobilized polymeric nanoparticles
Source: Sci Technol Adv Mater. 2025 Feb 6;26(1):2463316. doi: 10.1080/14686996.2025.2463316 (PMC12551373; doi:10.1080/14686996.2025.2463316)
Supplement: Supplemental Material [file TSTA_A_2463316_SM6356.pdf]

Supporting Information

Exploring anti-cancer activities of epidermal growth factor-immobilized polymeric nanoparticles

Shota Yamamoto[a]*, Chia-Jung Chang[a], Masao Kamimura[b], and Jun Nakanishi[a,b,c,d]*

[a] Research Center for Macromolecules & Biomaterials, National Institute for Materials Science (NIMS), 1-1 Namiki, Tsukuba, Ibaraki 305-0044, Japan; [b] Graduate School of Advanced Engineering, Tokyo University of Science, 6-3-1 Niijuku, Katsushika-ku, Tokyo 125-8585, Japan; [c] Graduate School of Advanced Science and Engineering, Waseda University, 3-4-1 Okubo, Shinjuku-ku, Tokyo 169-8555, Japan; [d] Research Center for Autonomous Systems Materialogy (ASMat), Institute of Integrated Research (IIR), Institute of Science Tokyo (Science Tokyo), 4259 Nagatsuta-cho, Midori-ku, Yokohama, Kanagawa 226-8501, Japan.

Corresponding authors: YAMAMOTO.Shota@nims.go.jp (S.Y); NAKANISHI.Jun@nims.go.jp (J.N).

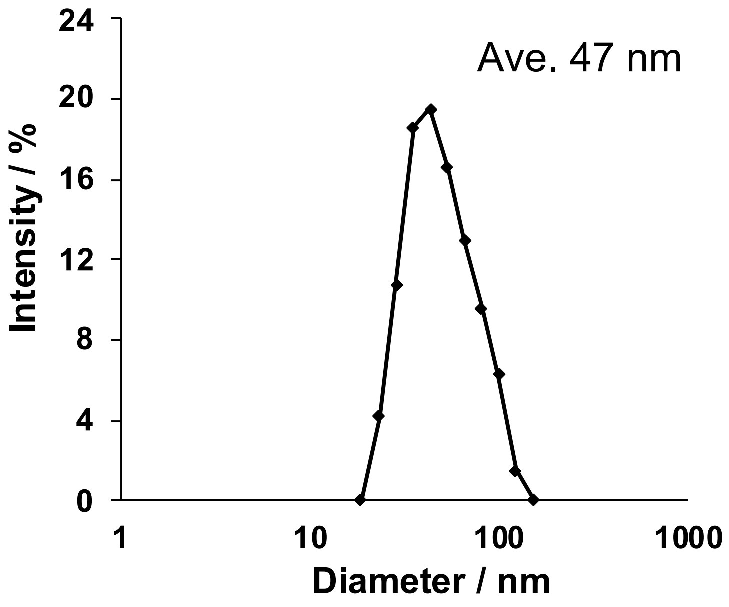

Figure S1. DLS result of 50 nm PEG-PSNPs without EGF.

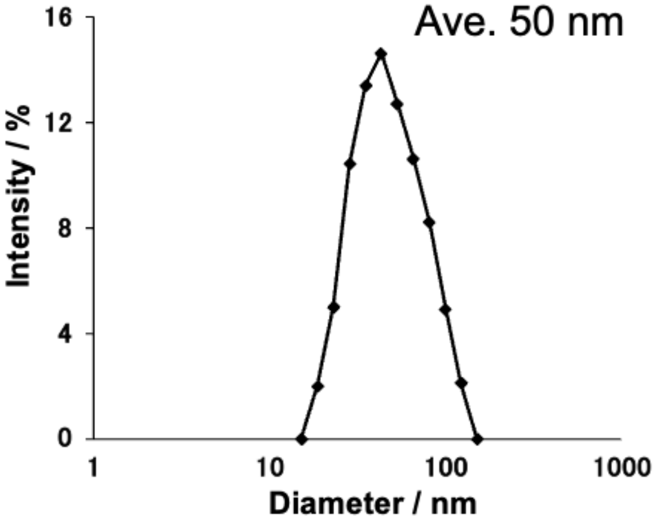

Figure S2. Dynamic light scattering analysis of EGF-polymeric micelles three days after preparation.

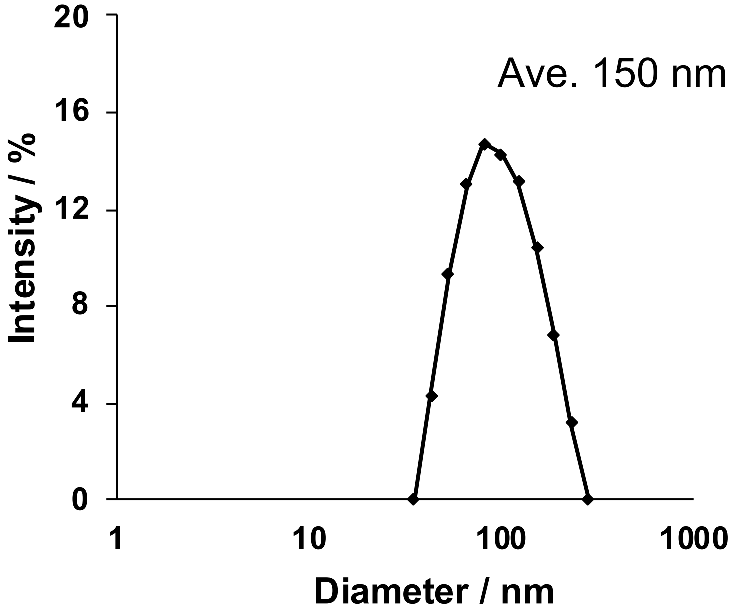

Figure S3. DLS result of 150 nm EGF-polymeric micelles.

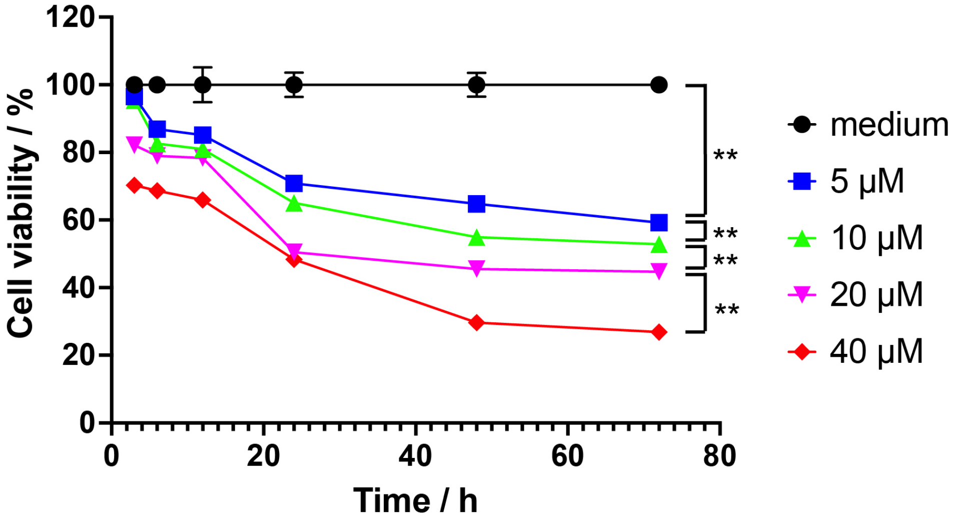

Figure S4. The dose dependent curves of growth inhibitory activity of 150 nm EGF-polymeric micelles. MDA-MB468 cells were treated with EGF-polymeric micelles for 72 hours. Cell viability was calculated based on the growth inhibitory effects relative to those treated with medium. Each data represents mean ± SD from at least three independent experiments. Statistical difference was evaluated by Student's *t*-test (***P* < *0.01)*.

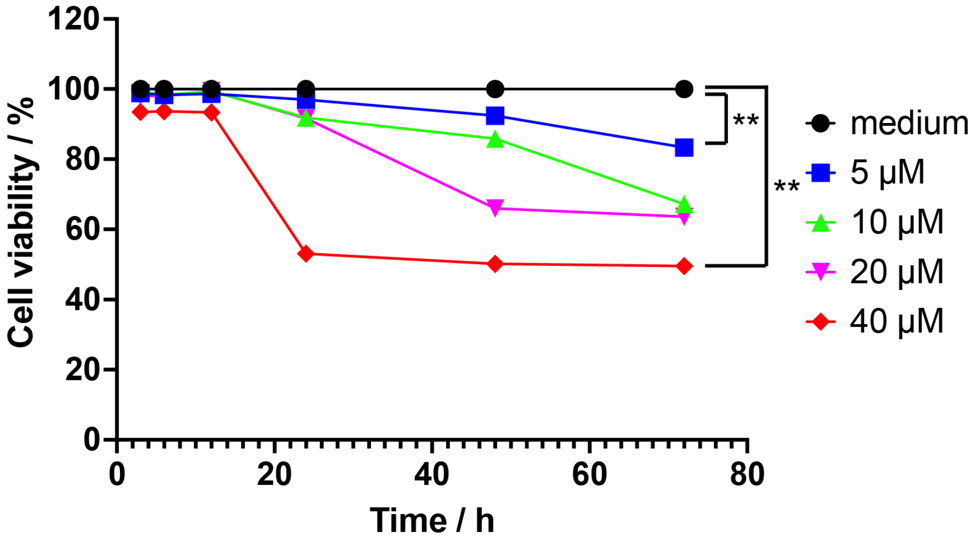

Figure S5. The dose dependent curves of growth inhibitory activity of 150 nm polymeric micelles without EGF. MDA-MB468 cells were treated with polymeric micelles without EGF. Cell viability was calculated based on the growth inhibitory effects relative to those treated with medium. Each data represents mean ± SD from at least three independent experiments. Statistical difference was evaluated by Student's *t*-test (**$P < 0.01$).

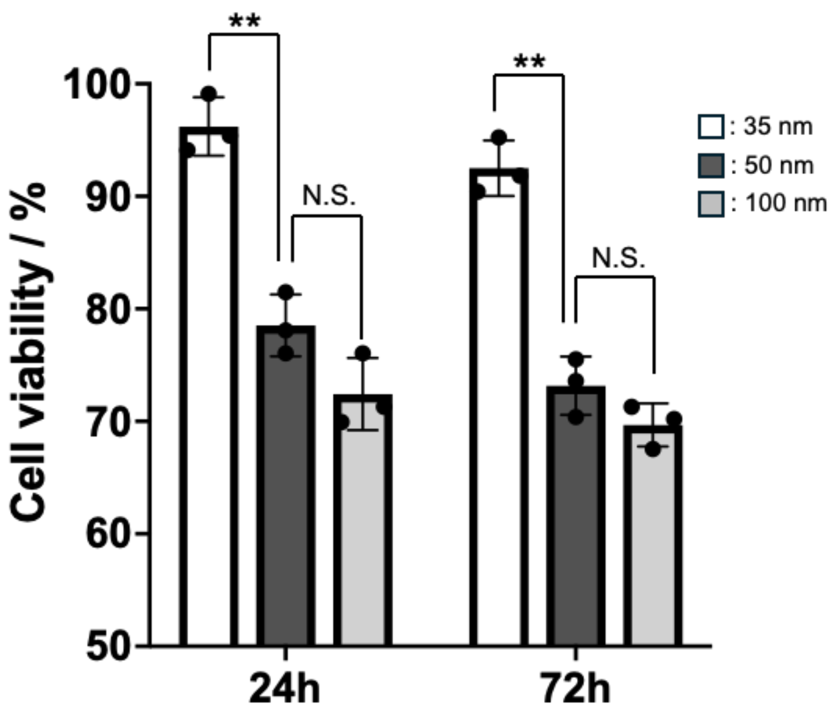

Figure S6. The size dependent of growth inhibitory activity of 35 nm, 50 nm and 100 nm EGF-polymeric micelles (15 μM). Cell viability was calculated based on the growth inhibitory effects relative to those treated with medium. Each data represents mean ± SD from at least three independent experiments. Statistical difference was evaluated by Student's *t*-test (*$**P < 0.01$*).

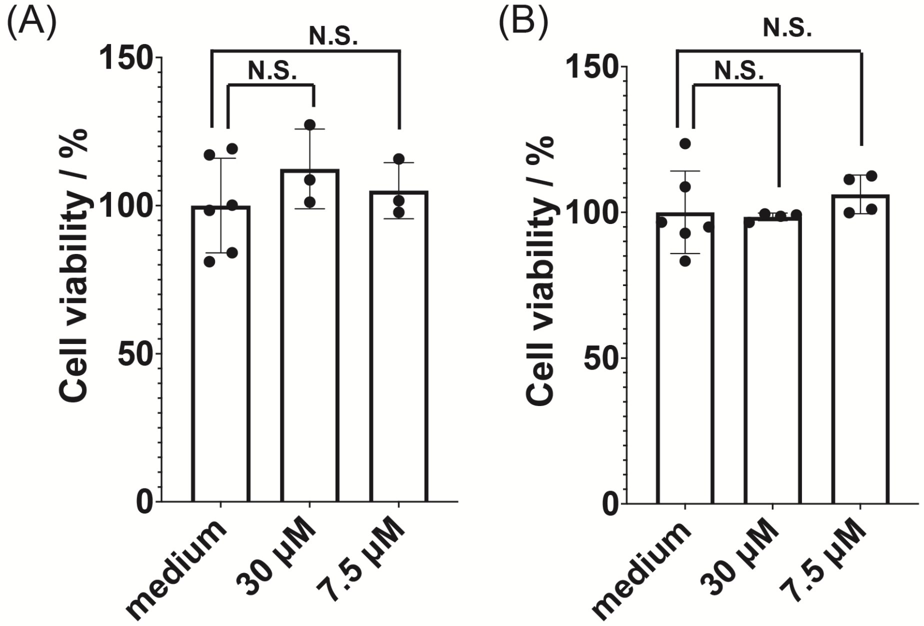

Figure S7. Growth inhibitory activity of 50 nm EGF-polymeric micelles. MCF10A cells were treated either with EGF-polymeric micelles for (A) 24 hours and (B) 72 hours. Cell viability was calculated based on the growth inhibitory effects relative to those treated with medium. Each data represents mean ± SD from at least three independent experiments. Statistical difference was evaluated by Student's *t*-test (*$**P < 0.01$*).

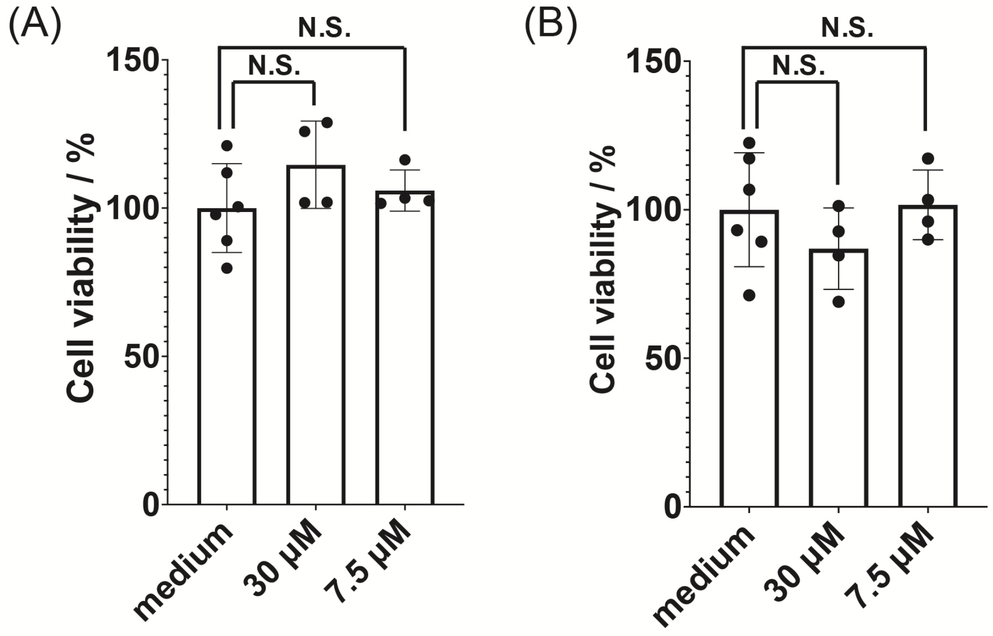

Figure S8. Growth inhibitory activity of 50 nm EGF-polymeric micelles. EA.hy926 cells were treated either with EGF-polymeric micelles for (A) 24 hours and (B) 72 hours. Cell viability was calculated based on the growth inhibitory effects relative to those treated with medium. Each data represents mean ± SD from at least three independent experiments. Statistical difference was evaluated by Student's *t*-test (*$^{**}P < 0.01$*).
